# Micromorphology of Labellum in Selected *Dendrobium* Sw. (Orchidaceae, Dendrobieae)

**DOI:** 10.3390/ijms23179578

**Published:** 2022-08-24

**Authors:** Aleksandra Burzacka-Hinz, Magdalena Narajczyk, Magdalena Dudek, Dariusz L. Szlachetko

**Affiliations:** 1 Department of Plant Taxonomy and Nature Conservation, Faculty of Biology, University of Gdańsk, Wita Stwosza 59, 80-308 Gdańsk, Poland; 2 Laboratory of Electron Microscopy, Faculty of Biology, University of Gdańsk, Wita Stwosza 59, 80-308 Gdańsk, Poland

**Keywords:** *Dendrobium*, labellum micromorphology, orchids, scanning electron microscopy, taxonomy

## Abstract

*Dendrobium* is one of the most species-rich genera of the Paleotropical orchids. It embraces more than 1000 species, most of which are epiphytes. The strong variation in floral characters causes many identification difficulties within this genus. One of the key structures, often sufficient in identification on a species level, is the labellum, which in many species of *Dendrobium* possesses a thickened callus and various types of trichomes and papillae. The aim of this study is to identify and describe the structures present on the labellum surface of the analyzed species, determine their distribution and density, as well as to check whether the obtained data have taxonomic value. In this paper, we present the results of a micromorphological study on the labellum of 21 species of *Dendrobium*, representing 13 sections, using scanning electron microscopy (SEM). Our studies revealed the presence of both uni- and multicellular structures on the surface of the labellum. We observed three types of trichomes (conical, cylindrical, ellipsoidal) and three types of papillae (conical, cylindrical, semicircular). Neither trichomes nor papillae were recorded for five species. In addition, we made diagrams showing the distribution and density of structures on the labellum. Based on the micromorphological results combined with the phylogenetic tree performed, we suggest that the presence/absence of labellum structures does not necessarily reflect the phylogenetic relationship and might be misleading, as in some cases, they arise due to convergence.

## 1. Introduction

*Dendrobium* Sw. was described by Olof Swartz in 1799 [1]. Currently, it is one of the largest genera belonging to the Orchidaceae, with over 1000 species [2]. They can grow as terrestrial plants or lithophytes, although most of them are epiphytes. Their range extends from Sri Lanka and India throughout tropical Asia and Oceania, north to Japan, east to Tahiti, and south to New Zealand [3].

The representatives of this genus are characterized by the presence of a lateral inflorescence arising from the upper part of the pseudobulbs. The flowers can reach from 0.4 cm to 17 cm in diameter and vary in form and color [4]. Distinctive features are also the presence of a mentum formed by lateral sepals and the column foot, and the presence of four naked, laterally compressed pollinia [4,5]. The mentum is usually slim and forms a spur-like structure, sometimes relatively long. In most species, the labellum is three-lobed and consists of a middle lobe and two lateral lobes. Usually, the middle lobe is tufted and flat in front, tapering towards the rear. At the base of the labellum, there are usually protrusions called callus, hairs, and various types of lumps. These structures, arranged in characteristic patterns, can lure and guide visiting pollinators, most often bees. Unfortunately, there is still little information on pollination of the *Dendrobium* species. Many species of *Dendrobium* are morphologically very similar to each other, however, some species have overlapping morphological differences that may result in misidentifications [6,7]. In this case, the classical methods of identification that rely on distinguishing characteristic features visible to the naked eye are not sufficient.

Probably the lack of exhaustive data may be due to the sparse studies on the micromorphology of the labellum within this genus; many works facing a similar problem have been published, but they concerned other groups of orchids [8,9,10,11,12]. Only the results obtained by Davies showed that labellum-trichomes and pseudopollen are presented on *Dendrobium unicum* Seidenf [13]. The term pseudopollen most often denotes a floury, yellow–white material, usually, but not always, containing nutrients and externally resembling pollen [10,13]. Often the presence of food is intended to reward visiting pollinators. However, a lack of nutrients does not equal a lack of attracting insects, and species can still do this thanks to mimicry.

The trichomes of *Dendrobium unicum* differ from the trichomes of other orchid representatives, such as *Maxillaria* or *Polystachya*. The differences concern the way they are formed or the presence of various nutrients. In *Dendrobium unicum*, the pseudopollen trichomes consist of a stalk cell, the head of component cells that separate at maturity, and their main food substance is starch. In contrast, in members of *Maxillaria*, pseudopollen is formed by the fragmentation of single-row hairs, resulting in the formation of single cells or short chains of cells [13]. We can observe a similar situation in species of the nominal section of *Polystachya*. However, the feeding hairs found in the species from other sections of this genus are usually one or four-celled [10,13].

In this paper, we analyzed and recognized structures of the labellum from the selected representatives of *Dendrobium* using the scanning electron microscope (SEM). The aim of our studies was to identify and define the micromorphological structures which are potentially present. Moreover, we determined the variability of their distribution in the particular parts of the labellum.

Due to the large number of species of *Dendrobium*, the wide range of their occurrence, and also their huge morphological diversity, there are a lot of problems with the identification of species, and thus with intra-generic classification. Therefore, the micromorphological studies of the labellum of the genus representatives can also be a valuable source of taxonomic information. In addition, they will constitute the foundation for further research on the pollination of *Dendrobium* flowers.

## 2. Results

### 2.1. SEM

**Figure 1:**
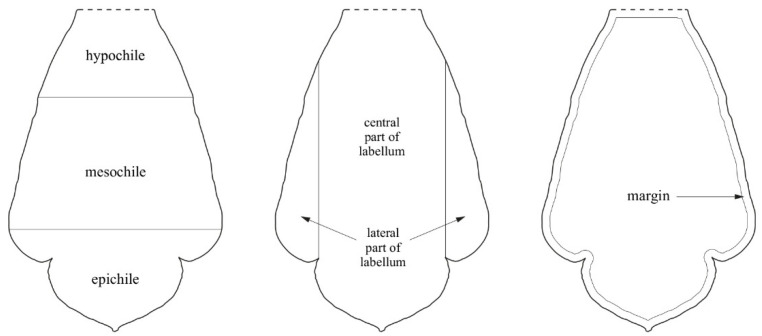
Terminology for parts of the labellum used in the Results section.

While describing the labellum and determining the distribution of the analyzed structures, the terminology presented in Figure 1 was used. The following parts of the labellum were distinguished: the basal part, the hypochile; the apical part, the epichile; and the section between the two aforementioned, the mesochile.

Two characteristic forms can be observed on the surface of the examined lips: papillae and trichomes. The first ones are single-celled, with small protrusions of various lengths, usually wider at the base [10,14]. The trichomes are unicellular or multicellular structures of various shapes with a narrow point of insertion and often with a differently shaped apex [10].

**Figure 2:**
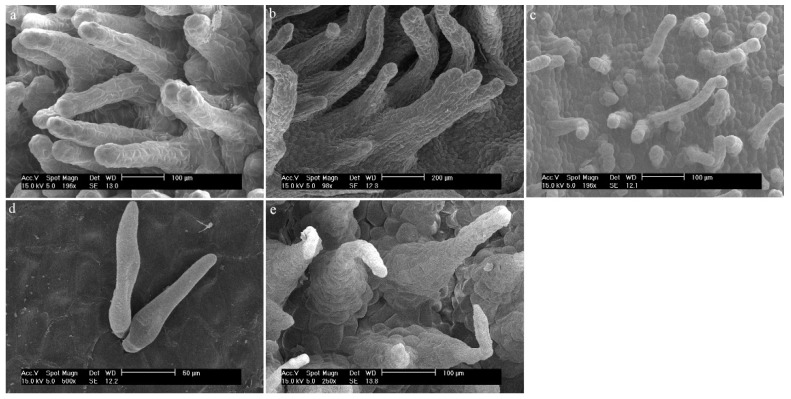
The types of trichomes found on the labellum of the *Dendrobium* species: (**a**) cylindrical; (**b**) cylindrical and branched; (**c**) cylindrical with a bulbous cell at the apex; (**d**) ellipsoidal; (**e**) conical.

**Figure 3:**
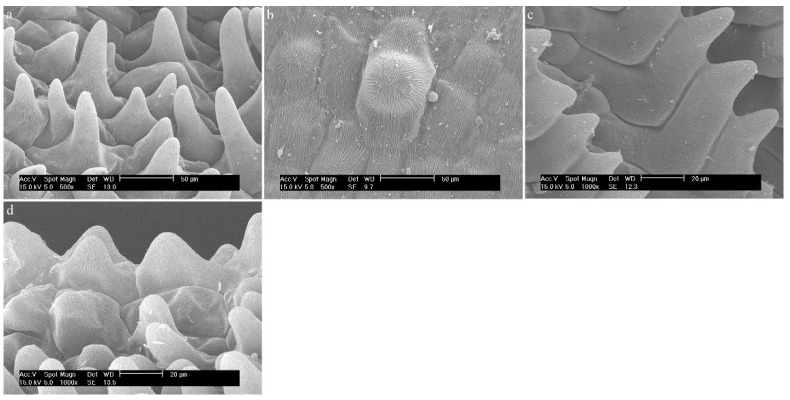
The types of papillae found on the labella of the *Dendrobium* species: (**a**) cylindrical and conical; (**b**) semicircular; (**c**) conical; (**d**) conical with rounded or pointed apices.

Three different types of papillae and three different types of trichomes have been observed. All types that appeared in this review are presented below (Figure 2 and Figure 3, Table 1). The descriptions also considered the shape of the epidermis, bearing, among others, trichomes and papillae, and the presence of callus-protuberances found in some species [14].

**Figure 4:**
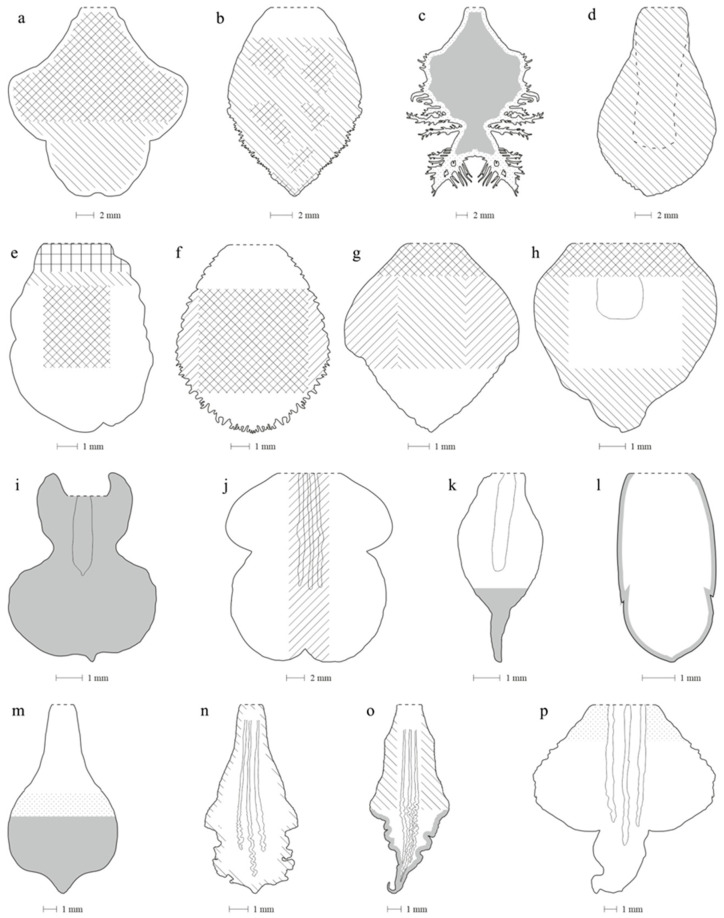
Relative density of trichomes and papillae on the labellum of *Dendrobium*: (**a**) *D. hainanense*; (**b**) *D. anosmum*; (**c**) *D. brymerianum*; (**d**) *D. chrysocrepis*; (**e**) *D. nobile*; (**f**) *D. parishii*; (**g**) *D. regium*; (**h**) *D. scoriarum*; (**i**) *D.* × *delicatum*; (**j**) *D. christyanum*; (**k**) *D. katherinae*; (**l**) *D. monophylum*; (**m**) *D. cuthbertsonii*; (**n**) *D. mortii*; (**o**) *D. schoeninum*; (**p**) *D. discolor*.

Due to the different organization of the papillae and trichomes, labella were drawn (with appropriate dimensions), which schematically show their distribution and density. The juxtaposed labella are shown in Figure 4, and the compaction intensity scale used in the drawings is presented in Table 2.

The descriptions of the investigated species according to sections are presented below. The first part includes species with labellar protrusions, and the second part includes species without trichomes and papillae.

### 2.2. Species with Labellar Protrusions

#### 2.2.1. *Dendrobium* Sw. sect. *Aporum* Blume

**Figure 5:**
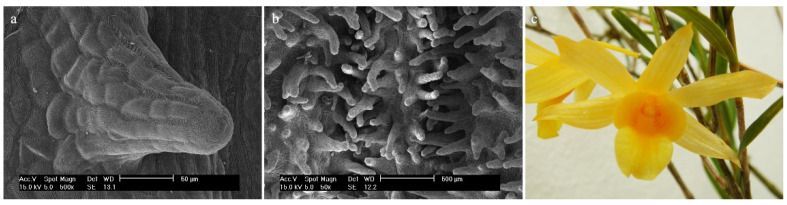
*Dendrobium hainanense* Rolfe.: trichomes (**a**) of the hypochile part of the labellum; (**b**) of the epichile part of the labellum; (**c**) flower (photo by Monika Lipińska).

1. *Dendrobium hainanense* Rolfe
  On the entire surface of the labellum, there are trichomes covered with a folded cuticle (Figure 5a). In the hypochile and the mesochile, the raised epidermal cells (column-shaped) are single and small. There are conical-shaped trichomes that are rare (Figure 5a). The epichile has much fewer individual, multicellular trichomes (epidermal cells) that vary in size (Figure 5b). Trichomes are branched and multicellular, most often cylindrical in shape and numerous (Figure 5b). The edges of the labellum are smooth, without trichomes.

#### 2.2.2. *Dendrobium* Sw. sect. *Dendrobium*

**Figure 6:**
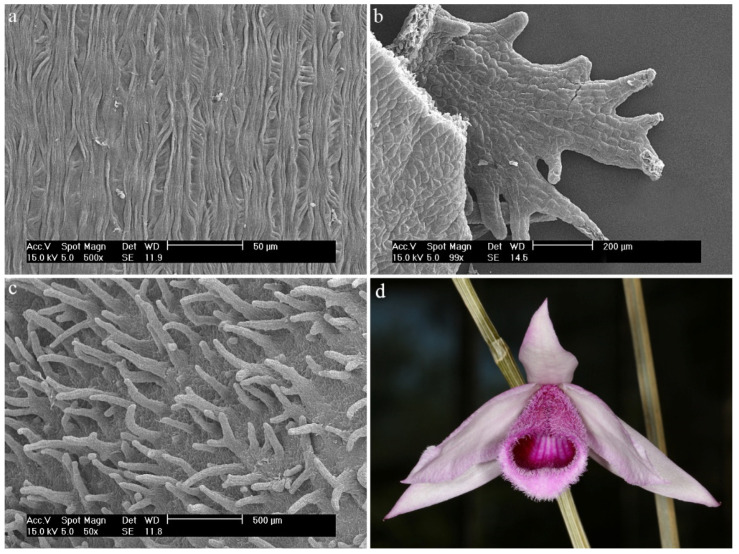
*Dendrobium anosmum* Lindl.: (**a**) the hypochile part of the labellum; (**b**) margin of the mesochile part of the labellum; (**c**) trichomes of the epichile part of the labellum; (**d**) flower (photo by John Varigos).

**Figure 7:**
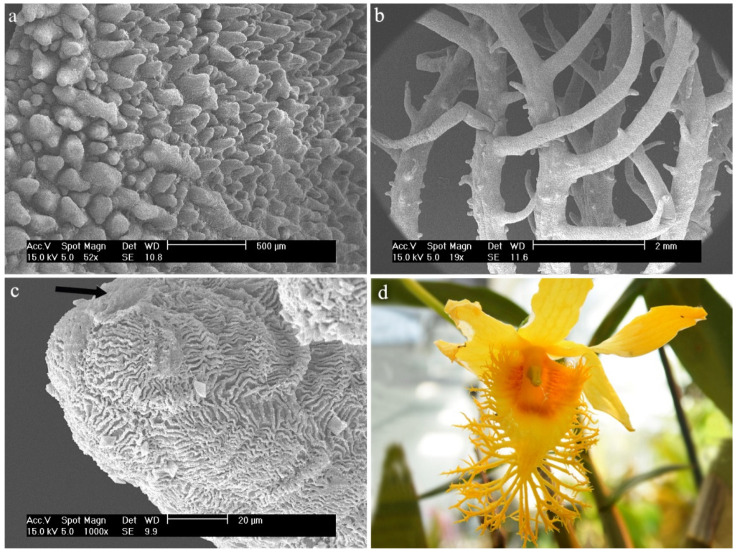
*Dendrobium brymerianum* Rchb.f.: trichomes (**a**) of the mesochile part of the labellum; (**b**) of the margin of the mesochile part of the labellum; (**c**) of the epichile part of the labellum; (**d**) flower (photo by Monika Lipińska).

**Figure 8:**
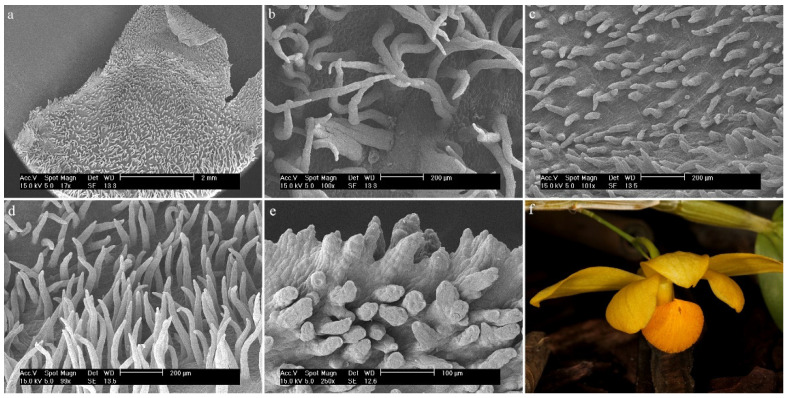
*Dendrobium chrysocrepis* C.S.P.Parish & Rchb.f. ex Hook.f.: trichomes (**a**) of the mesochile and the epichile part of the labellum; (**b**) of the final part of the hypochile of the labellum/initial part of the mesochile of the labellum; (**c**) of the mesochile part of the labellum; (**d**) of the epichile part of the labellum; (**e**) of the margin of the epichile part of the labellum; (**f**) flower (photo by John Varigos).

**Figure 9:**
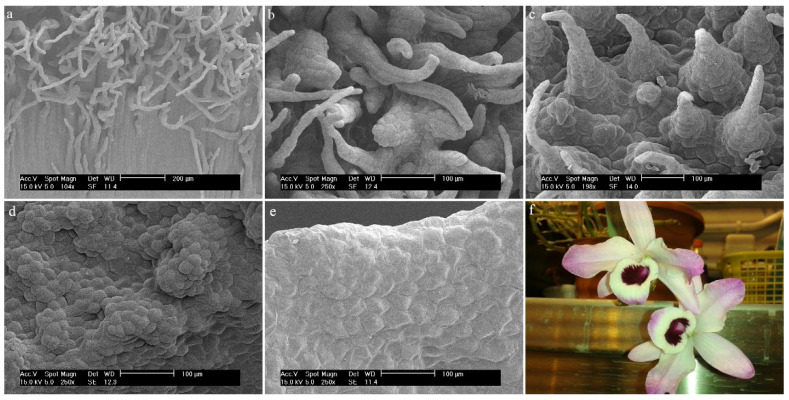
*Dendrobium nobile* Lindl: (**a**,**b**) trichomes of the hypochile part of the labellum; (**c**) trichomes of the mesochile part of the labellum; (**d**) margin of the mesochile part of the labellum; (**e**) margin of the epichile part of the labellum; (**f**) flower (photo by Monika Lipińska).

**Figure 10:**
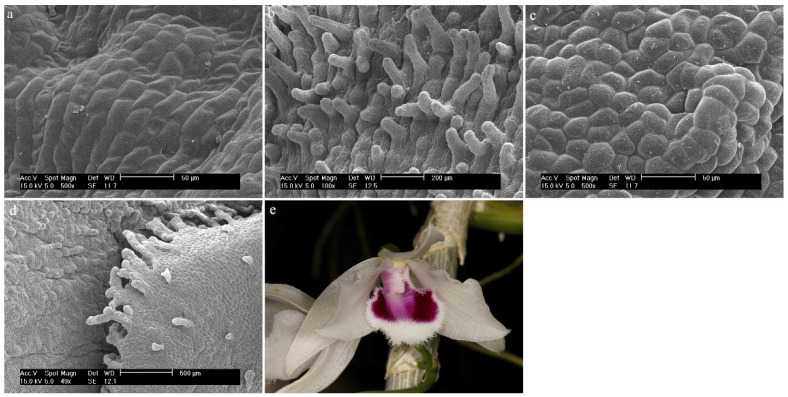
*Dendrobium parishii* H.Low: (**a**) the hypochile part of the labellum; (**b**) trichomes of the mesochile part of the labellum; (**c**) margin of the mesochile part of the labellum; (**d**) trichomes of the margin of the mesochile part of the labellum; (**e**) flower (photo by John Varigos).

**Figure 11:**
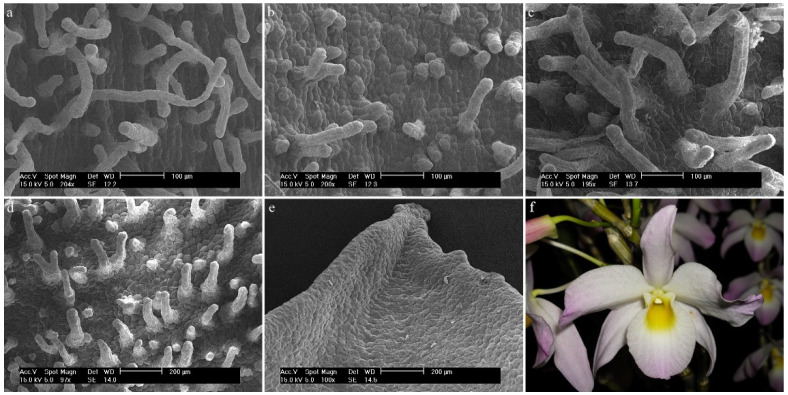
*Dendrobium regium* Prain: (**a**) trichomes of the hypochile part of the labellum; (**b**) trichomes of the final part of the hypochile of labellum/initial part of the mesochile of the labellum; (**c**) trichomes of the mesochile part of the labellum; (**d**) trichomes of the margin of the mesochile part of the labellum; (**e**) the epichile part of the labellum; (**f**) flower (photo by John Varigos).

**Figure 12:**
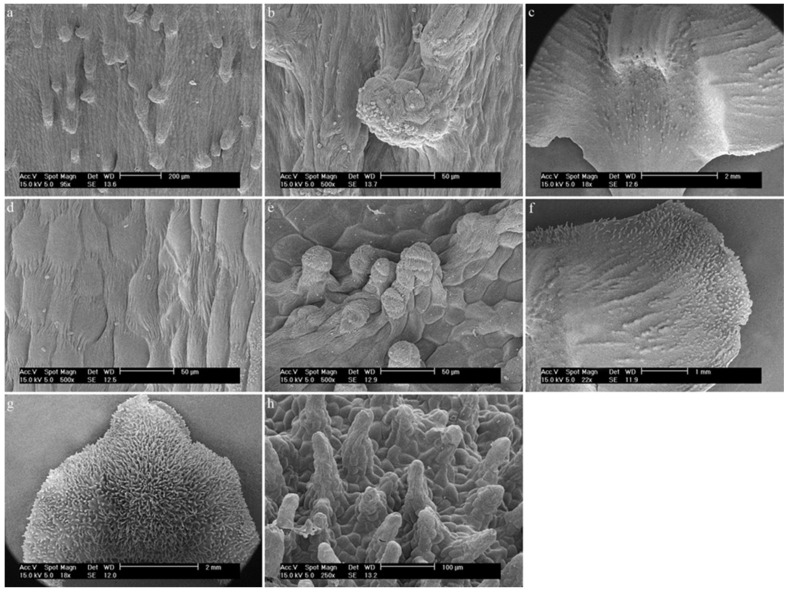
*Dendrobium scoriarum* W.W.Sm.: (**a**) the hypochile part of the labellum; (**b**) trichome of the hypochile part of the labellum; (**c**,**d**) the final part of the hypochile of the labellum/initial part of the mesochile of the labellum; (**e**) trichomes of the margin of the mesochile part of the labellum; (**f**) the mesochile part of the labellum; (**g**,**h**) trichomes of the epichile part of the labellum.

1. *Dendrobium anosmum* Lindl.
  The hypochile consists of epidermal cells covered with a thick, folded cuticle. The folding is regular and extends over the entire surface of the labellum (Figure 6a). In the mesochile and the epichile, epidermal cells are regular and smooth, forming a jagged structure at the edges (Figure 6b) and multicellular conical and cylindrical trichomes, sometimes bifurcated at the ends (Figure 6c). In some parts of the surface, their occurrence can be described as rare, but there are also places where their density is higher. The cuticle is smooth.

2. *Dendrobium brymerianum* Rchb.f.
  The entire surface of the labellum is filled with numerous conical and semicircular papillae. The hypochile has lower papillae than the mesochile and the epichile (Figure 7a). A cuticle is unseen. The edges of the labellum are jagged and branched (Figure 7b). They are made up of epidermal cells covered with a very strongly folded cuticle (Figure 7c). The secretion is possible at the very apex (Figure 7c, marked with an arrow).

3. *Dendrobium chrysocrepis* C.S.P.Parish & Rchb.f. ex Hook.f.
  The entire surface of the labellum is densely covered with conical trichomes (Figure 8a). The final part of the hypochile/initial part of the mesochile is formed by regular epidermal cells with elongated trichomes that are clustered in pairs or appear in a set of three (Figure 8b). In the middle part of mesochile, epidermal cells are slightly elongated, forming multicellular trichomes with a slightly folded cuticle (Figure 8c). The epichile is made up of epidermal cells, which also form elongated and multicellular trichomes (the longest on the entire labellum) (Figure 8d), although they are low at the edges (Figure 8e). A cuticle is unseen.

4. *Dendrobium nobile* Lindl.
  The hypochile is composed of elongated epidermal cells forming longitudinal, multicellular cylindrical and conical trichomes, which in some places are so densely distributed that their apical part is often not visible (Figure 9a,b). The surface of the mesochile is made up of regular epidermal cells, forming sparsely distributed, multicellular conical trichomes. Trichomes at the base are very wide, tapering towards the apex (Figure 9c). The sides of this part are filled with regular epidermal cells, covered with a folded cuticle (epidermal cells form hills) without any trichomes or papillae (Figure 9d). There are also no trichomes in the terminal epichile part (Figure 9e).

5. *Dendrobium parishii* H.Low
  The hypochile has regular epidermal cells without trichomes (Figure 10a). The mesochile also consists of regular epidermal cells, which in the central part form rarely arranged regular, multicellular cylindrical trichomes (Figure 10b). The cuticle is rather smooth. The margin of the mesochile part of the labellum is made up of regular epidermal cells (Figure 10c) that form irregular and jagged edges (Figure 10d). The cuticle here is very slightly folded (Figure 10c). There are single trichomes here, but in the apical part of the epichile, none of them are visible.

6. *Dendrobium regium* Prain
  The hypochile is made up of regular epidermal cells. Rarely, there are elongated, multicellular cylindrical trichomes with a bulbous cell at the apex (Figure 11a). Further on, regular epidermal cells form lower, multicellular, and more cylindrical trichomes (Figure 11b). The mesochile, like the hypochile, consists of regular epidermal cells and has elongated cylindrical trichomes ending with a bulbous cell, which are abundant here (Figure 11c). In this part, both sides of the labellum are also composed of regular epidermal cells with multicellular trichomes, but lower than the trichomes in the middle part and cylindrical, occurring in a small number (Figure 11d). The epichile is composed of regular and smooth epidermal cells; it has no trichomes (Figure 11e).

7. *Dendrobium scoriarum* W.W.Sm.
  The hypochile is composed of elongated epidermal cells (Figure 12a) that form parallel, multicellular cylindrical trichomes covered with a folded cuticle (Figure 12b). They are rare in this part. Then, the visible callus is made up of regular epidermal cells with a cuticle, which is folded at the junctions between the cells (Figure 12c,d). In the mesochile part, the edges of the labellum are made up of regular epidermal cells, which are only elongated at the site of the formation of multicellular conical and cylindrical trichomes, which occur numerously there (Figure 12e). In the central part of the mesochile, the cuticle covering the epidermis is folded and there are no trichomes on it (Figure 12f). The entire epichile surface is densely covered with multicellular trichomes, mostly conical and sometimes cylindrical (Figure 12g,h). The epidermal cells are regular.

#### 2.2.3. *Dendrobium* Sw. sect. *Dendrocoryne* Lindl. & Paxton

**Figure 13:**
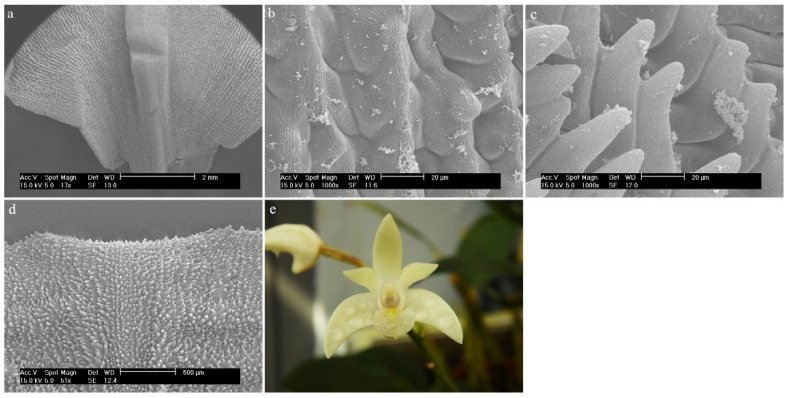
*Dendrobium* × *delicatum* (F.M.Bailey) F.M.Bailey: papillae (**a**) of the hypochile and the mesochile part of the labellum; (**b**) of the hypochile part of the labellum, (**c**) of the mesochile part of the labellum; (**d**) of the epichile part of the labellum; (**e**) flower (photo by Monika Lipińska).

1. *Dendrobium* × *delicatum* (F.M.Bailey) F.M.Bailey
  Throughout the center of the hypochile and the mesochile parts, a fold-like callus extends, which is raised above the remaining cells. There are densely arranged semicircular papillae resembling a dragon’s scale. They are lower than the papillae outside the fold. The papillae outside the fold appear in rows, one after the other (Figure 13a), also resembling the scales of a dragon (Figure 13b). The closer to the epichile, the longer the papillae are, covered with a slightly folded cuticle, and their shape is more conical (Figure 13c). There is possible secretion on the surface. The entire surface of the epichile also features densely arranged papillae with a slightly folded cuticle, conical in shape, and sometimes more cylindrical (Figure 13d).

#### 2.2.4. *Dendrobium* Sw. sect. *Formosae* (Benth. & Hook.f.) Hook.f.

**Figure 14:**
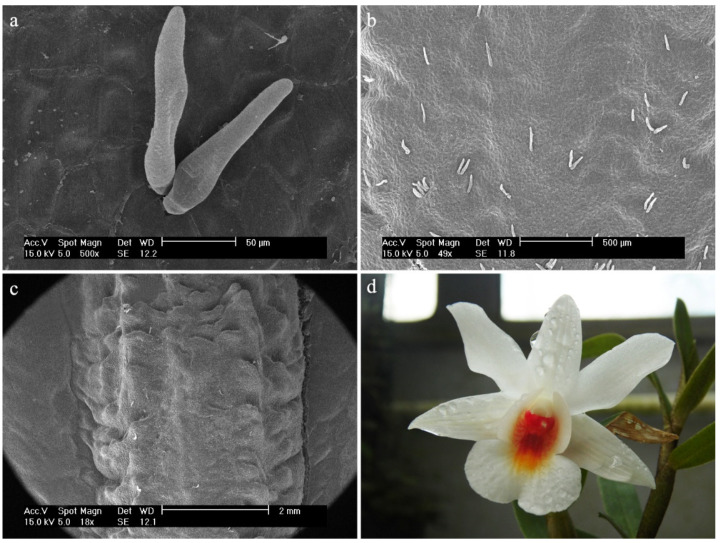
*Dendrobium christyanum* Rchb.f. Trichomes: (**a**,**b**) of the hypochile part of the labellum; (**c**) of the mesochile part of the labellum; (**d**) flower (photo by Monika Lipińska).

1. *Dendrobium christyanum* Rchb.f.
  Throughout the center of the labellum, there is a callus that consists of regular epidermal cells. The edges of the elevation are made up of epidermis cells resembling frills. In part of the hypochile, it is made up of typical, unextended epidermal cells covered with a rather delicately folded cuticle. The surface shows a few ellipsoidal single-celled trichomes with a smooth cuticle (Figure 14a,b), which are also present on the further parts of the elevation (Figure 14c).

#### 2.2.5. *Dendrobium* Sw. sect. *Grastidium* (Blume) Blume

**Figure 15:**
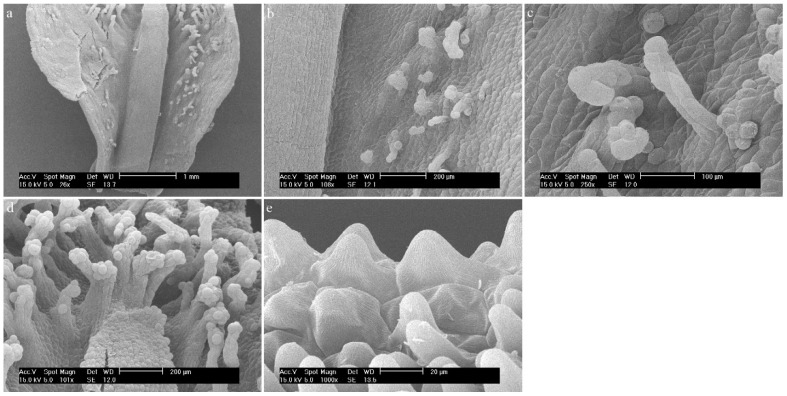
*Dendrobium katherinae* A.D.Hawkes: (**a**) the hypochile and the mesochile part of the labellum; (**b**) the hypochile part of the labellum; (**c**) the initial part of the mesochile of the labellum; (**d**) final part of the mesochile of the labellum; (**e**) papillae of the epichile part of the labellum.

1. *Dendrobium katherinae* A.D.Hawkes
  Smooth, regular epidermal cells forming a callus are present in the central part of the hypochile and the mesochile (Figure 15a,b). In the initial, lateral part of the mesochile, there are visible multicellular clusters of the epidermis elevated above the surface. Their apex is crowned with round cells (Figure 15c). There are also similar patterns further on, but they are larger (longer) than those described earlier (Figure 15d). The surface of the epichile is covered with tightly formed papillae with a slightly folded cuticle (Figure 15e). They have a conical shape with rounded or pointed apices and are densely arranged.

#### 2.2.6. *Dendrobium* Sw. sect. *Monophyllaea* Benth.

**Figure 16:**
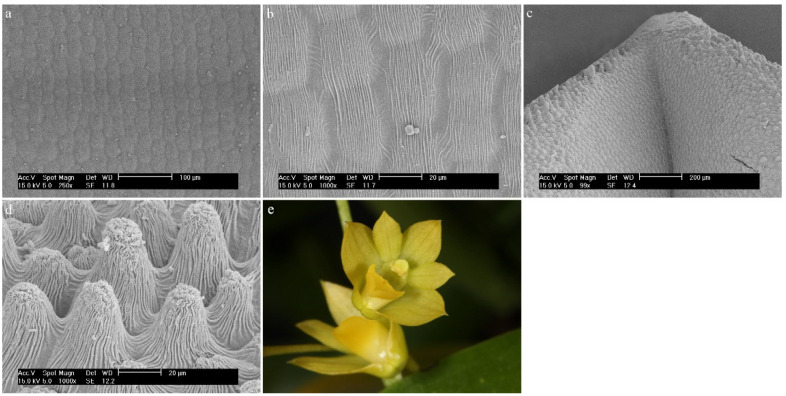
*Dendrobium monophyllum* F.Muell.: (**a**,**b**) the hypochile part of the labellum; (**c**) the mesochile and the epichile part of the labellum; (**d**) papillae of the margin of the epichile part of the labellum; (**e**) flower (photo by John Varigos).

1. *Dendrobium monophyllum* F.Muell.
  The labellum is made up of regular, elongated epidermal cells with visible cuticle folds (Figure 16a,b). On its edges, there are raised epidermal cells, which are probably densely arranged conical papillae with a strongly folded cuticle (Figure 16c,d).

#### 2.2.7. *Dendrobium* Sw. sect. *Oxyglossum* Schltr.

**Figure 17:**
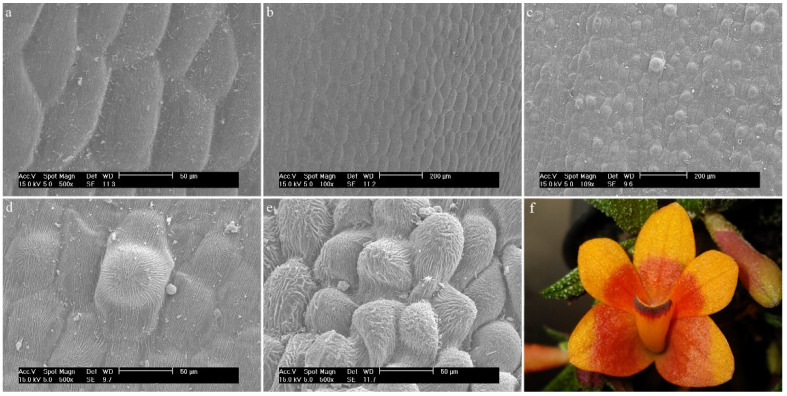
*Dendrobium cuthbertsonii* F.Muell.: (**a**,**b**) the hypochile part of the labellum; Papillae (**c**,**d**) of the mesochile part of the labellum; (**e**) of the epichile part of the labellum; (**f**) flower (photo by John Varigos).

1. *Dendrobium cuthbertsonii* F.Muell.
  The hypochile and the mesochile form epidermal smooth cells with a very delicately folded cuticle (Figure 17a,b). In the distal part of the mesochile, there are single semicircular papillae with a strongly corrugated cuticle (Figure 17c,d). The entire surface of the epichile is made up of densely arranged semicircular and conical papillae with a strongly folded cuticle (Figure 17e).

#### 2.2.8. *Dendrobium* Sw. sect. *Rhizobium* Lindl. & Paxton

**Figure 18:**
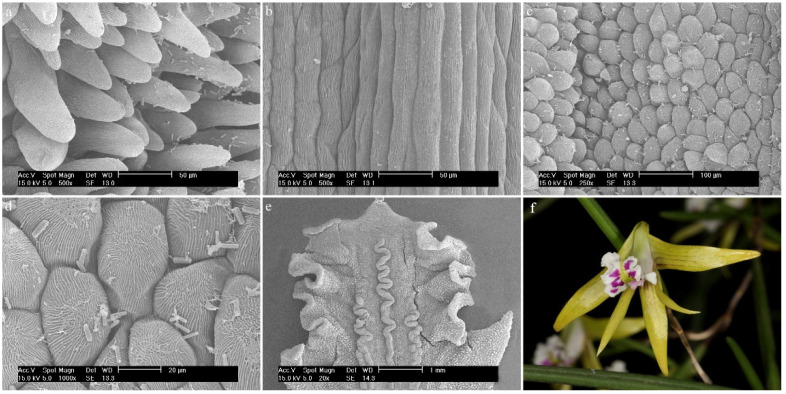
*Dendrobium mortii* F.Muell.: (**a**) trichomes of the hypochile part of the labellum; (**b**) the hypochile part of the labellum; (**c**,**d**) the mesochile part of the labellum; (**e**) the epichile part of the labellum with trichomes in the margin; (**f**) flower (photo by John Varigos).

**Figure 19:**
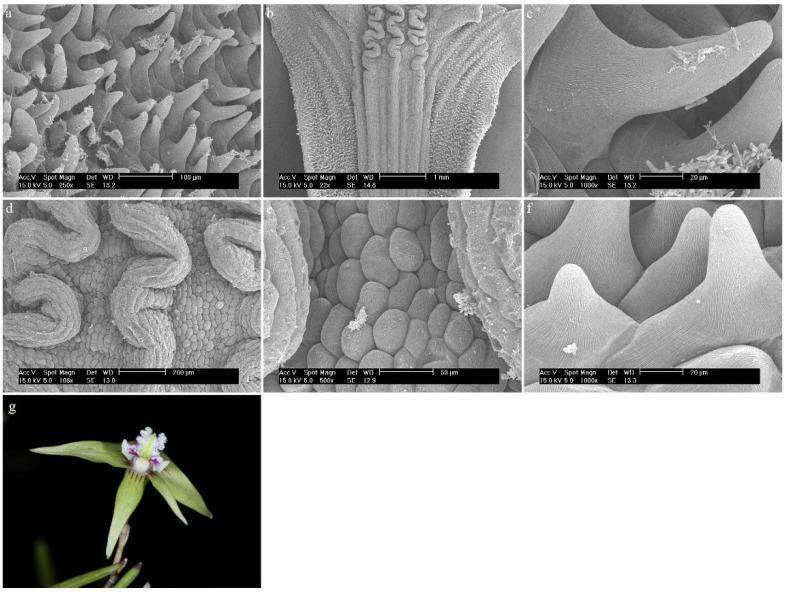
*Dendrobium schoeninum* Lindl.: (**a**) trichomes of the hypochile part of the labellum; (**b**,**c**) trichomes of the mesochile part of the labellum; (**d**,**e**) the mesochile part of the labellum; (**f**) papillae of the margin of the epichile part of the labellum; (**g**) flower (photo by John Varigos).

1. *Dendrobium mortii* F.Muell.
  On the edge of the whole labellum, there are densely arranged conical and cylindrical trichomes covered with a corrugated cuticle (Figure 18a). In the central part, there is a callus made up of elongated epidermal cells covered with a delicately folded cuticle. It forms three major furrows, which are folded further on, but the cells remain the same (Figure 18b). The central bulge is the longest. The next parts of the labellum are made up of slightly convex epidermal cells with a folded cuticle (Figure 18c). It is possible that there is secretion on their surface (Figure 18d). In the epichile part, on the margin, around the labellum, there are densely arranged trichomes of the same form, as in the part below (Figure 18e).

2. *Dendrobium schoeninum* Lindl.
  In the hypochile and the mesochile, on the lateral parts of the labellum, there are dense, tightly arranged conical trichomes covered with a delicately folded cuticle (Figure 19a–c). In the center, there is a callus made up of regular and elongated epidermal cells covered with a delicately folded cuticle. It forms three furrows that are present along the entire length of the labellum, but in its distal parts, they take a wavy form (Figure 19b,d). Between the folded furrows there are convex, oval epidermal cells with a slightly folded cuticle (Figure 19d,e). In the epichile part, the lateral edges are composed of densely arranged conical papillae with a folded cuticle (Figure 19f).

#### 2.2.9. *Dendrobium* Sw. sect. *Spatulata* Lindl.

**Figure 20:**
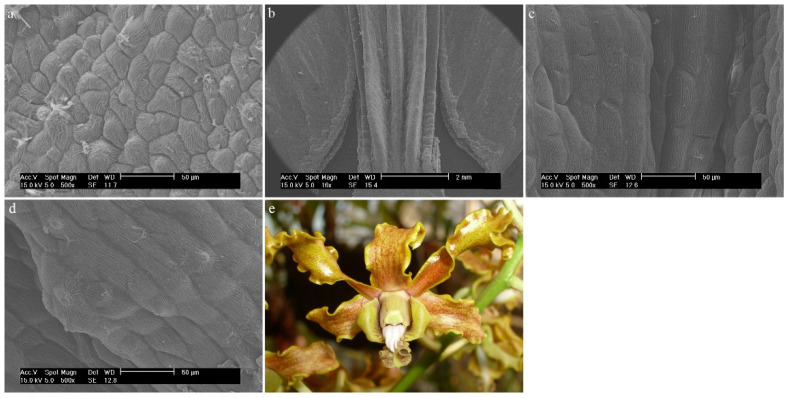
*Dendrobium discolor* Lindl.: (**a**) the epichile part of the labellum; (**b**) the hypochile and the mesochile part of the labellum; (**c**) the hypochile part of the labellum; (**d**) papillae of the hypochile part of the labellum; (**e**) flower (photo by John Varigos).

1. *Dendrobium discolor* Lindl.
  The entire surface of the labellum is composed of epidermal cells, covered with a strongly folded cuticle (Figure 20a). There are also protruding epidermal cells, forming a three-fold callus, extending from the hypochile to the epichile (Figure 20b). It is composed of elongated epidermal cells covered with a delicately folded cuticle (Figure 20c). In the hypochile part, there are a few small, conical-shaped papillae (Figure 20d).

### 2.3. Species with No Labellar Protrusions

#### 2.3.1. *Dendrobium* Sw. sect. *Conostalix* Kraenzl.

**Figure 21:**
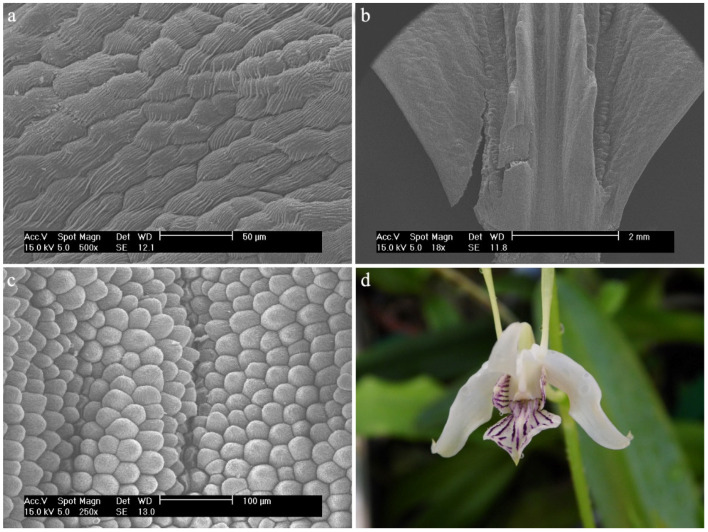
*Dendrobium attenuatum* Lindl.: (**a**) the mesochile part of the labellum; (**b**) the hypochile part of the labellum; (**c**) the epichile part of the labellum; (**d**) flower (photo by Monika Lipińska).

1. *Dendrobium attenuatum* Lindl.
  There are elongated epidermal cells with a folded cuticle on the entire labellum (Figure 21a). In the central part of the hypochile, the protruding portions of the labellum are visible, forming a callus reaching all the way to the tip (Figure 21b). The epichile may appear to have papillae, but they are elongated and convex epidermal cells (Figure 21c).

#### 2.3.2. *Dendrobium* Sw. sect. *Distichophyllae* Hk.f.

**Figure 22:**
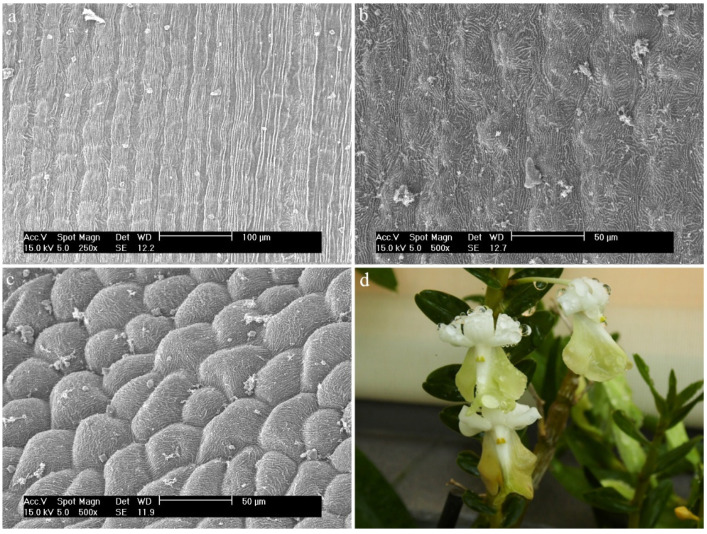
*Dendrobium uniflorum* Griff.: (**a**) the hypochile part of the labellum; (**b**) the mesochile part of the labellum; (**c**) the epichile part of the labellum; (**d**) flower (photo by Monika Lipińska).

1. *Dendrobium uniflorum* Griff.
  The entire surface of the labellum is free of trichomes and papillae. The hypochile is composed of elongated epidermal cells covered with a strongly folded cuticle (Figure 22a). The mesochile and the epichile are filled with delicately arched epidermal cells, covered with a very strongly folded cuticle (Figure 22b,c). (The fluff visible in the last part is not a secretion but dirt).

#### 2.3.3. *Dendrobium* Sw. sect. *Latouria* (Blume) Miquel

**Figure 23:**
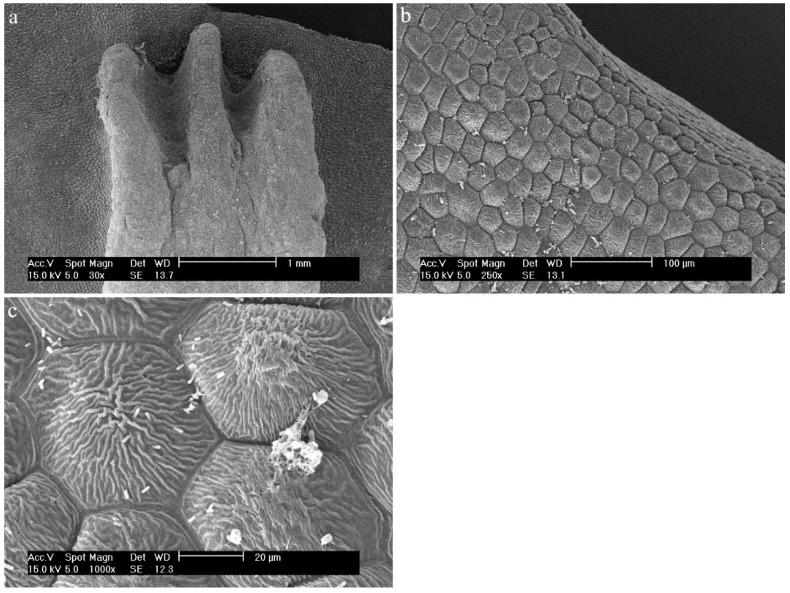
*Dendrobium bifalce* Lindl.: (**a**) the mesochile part of the labellum; (**b**,**c**) the epichile part of the labellum.

1. *Dendrobium bifalce* Lindl.
  In the central part of the labellum, there is a callus in the form of three combs, made up of elongated and smooth cells (Figure 23a). The sides of the labellum are composed of epidermal cells covered with a strongly folded cuticle (Figure 23b,c). The sticks visible on the surface are most likely to be bacteria.

#### 2.3.4. *Dendrobium* Sw. sect. *Pedilonum* (Blume) Blume

**Figure 24:**
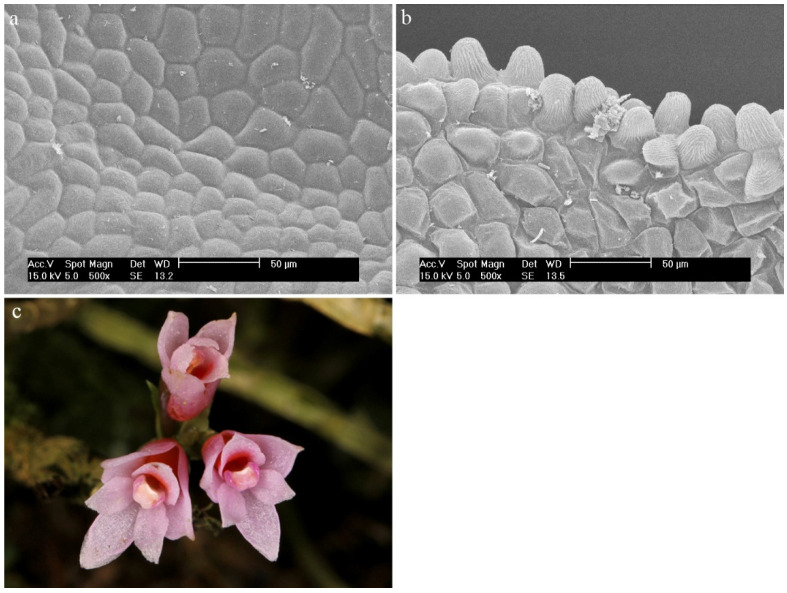
*Dendrobium alaticaulinum* P.Royen.: (**a**) the hypochile part of the labellum; (**b**) the mesochile part of the labellum; (**c**) flower (photo by John Varigos).

1. *Dendrobium alaticaulinum* P.Royen
  The labellum is made up of smooth, regular epidermal cells (Figure 24a). On its edges, there are slightly raised cells covered with a folded cuticle (Figure 24b).

#### 2.3.5. *Dendrobium* Sw. sect. *Spatulata* Lindl.

**Figure 25:**
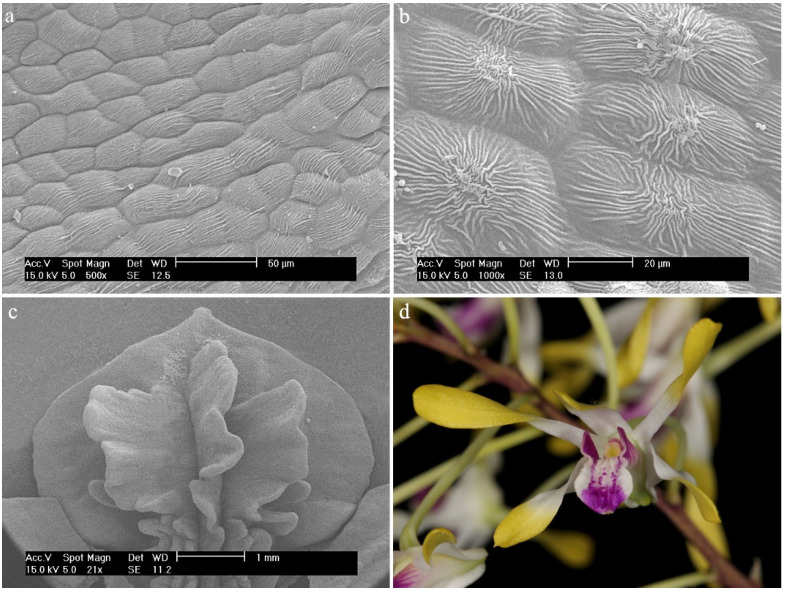
*Dendrobium canaliculatum* R.Br.: (**a**,**b**) the mesochile part of the labellum; (**c**) the epichile part of the labellum; (**d**) flower (photo by John Varigos).

1. *Dendrobium canaliculatum* R.Br.
  The entire labellum is made up of typical epidermal cells, covered with a folded cuticle (Figure 25a) that is gathered at the top of each cell (Figure 25b). In the central part of the labellum, a protruding callus is visible, which in the last part takes on a more undulating surface (Figure 25c). In the mesochile, on the lateral parts of the labellum, epidermal cells with possible secretion are visible (Figure 25a).

### 2.4. Phylogenetic Analysis

**Figure 26:**
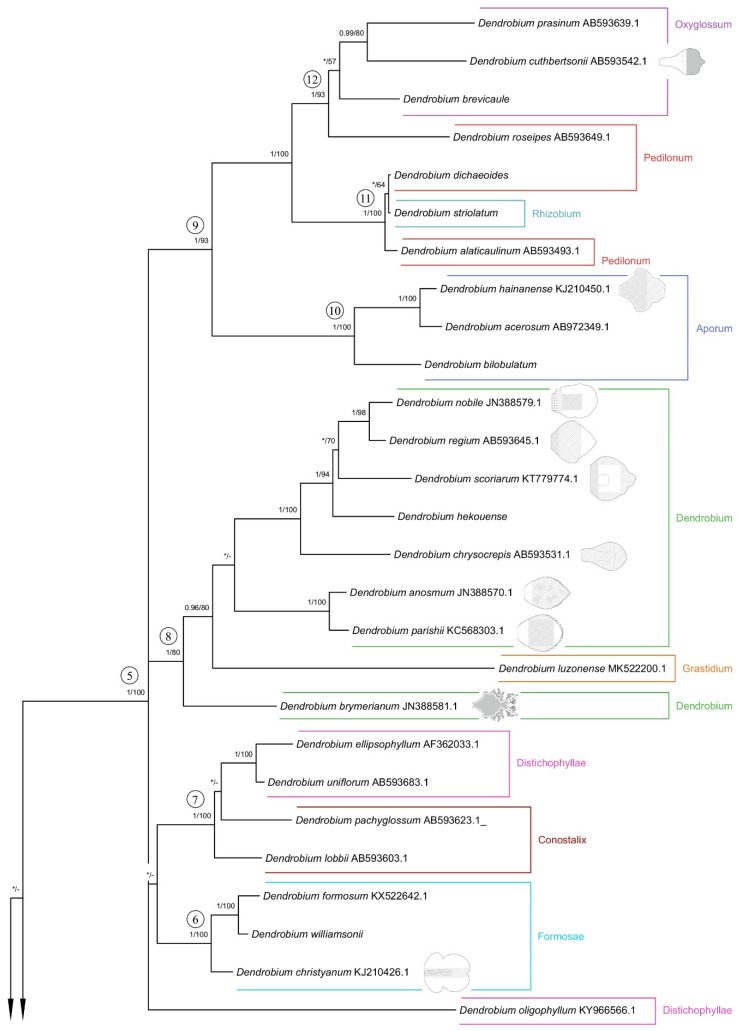
The 50% majority-rule consensus tree from the BI analysis of ITS marker. The numbers above the branches indicate posterior probability (PP) and bootstrap support (BS); PP < 0.95 were marked as an * and BS values of <50% were marked as -. Numbered black circles mark clades discussed in the text.

**Figure 27:**
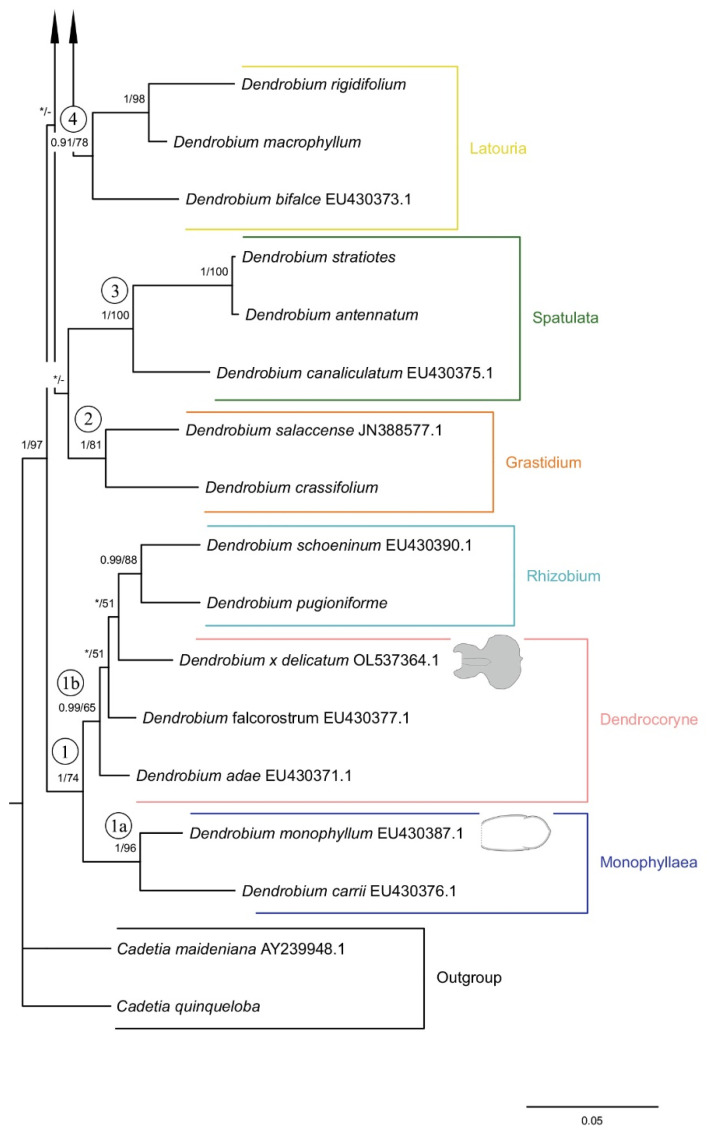
The 50% majority-rule consensus tree from the BI analysis of ITS marker (continuation of Figure 26). The numbers above branches indicate posterior probability (PP) and bootstrap support (BS); PP < 0.95 were marked as an * and BS values of <50% were marked as -. Numbered black circles mark clades discussed in the text.

Both applied methodologies (BI and MP) revealed the same results. The analyzed taxa grouped themselves into the same clades. We have noticed no differences in the topology of the trees obtained for the two methods used. Thus, we decided to present a 50% majority-rule consensus tree from the BI analysis (Figure 26 and Figure 27). However, we also placed bootstrap support values on the nodes for each clade. Representatives of the *Dendrobium* sect. *Rhizobium* ranked on the tree with species of the *Dendrocoryne* section (clade 1b) and with taxa of the *Pedilonum* section (clade 11). As a result of the analyses, the representatives of the recently mentioned section also did not form a monophyletic group. *Dendrobium roseipes* (sect. *Pedilonum*) joined together with species from section *Oxyglossum* (clade 12). We can observe a similar situation with taxa from section *Distichophyllae*. Two species of this section, *D. ellipsophyllum* and *D. uniflorum* formed a strongly supported clade 7 (PP = 1, BS = 100) with representatives of section *Conostalix*. In contrast, *D. oligophyllum* was placed at the base of clade 5 as a polytomic branching. It is worth noting that *D. luzonense* (sect. *Grastidium*) did not merge together with other members of the section (clade 2) only created one group with species of the nominal section (clade 8). However, the species of section *Monophyllaea* (clade 1a), *Spatulata* (clade 3), *Latouria* (clade 4), *Formosae* (clade 6), and *Aporum* (clade 10) have formed a strongly supported, monophyletic groups.

## 3. Discussion

Previous studies on the micromorphology structures of the representatives of *Dendrobium* have included only a few species of this genus [13,15]. In 1987, Kjellsson analyzed the labellum of *Dendrobium unicum* and, as in most cases in this study, also noted the presence of trichomes [15]. Although he did not show nutrients in them, he suggested that they might function as a pseudopollen. After extending these analyses and conducting histochemical studies, this fact was also confirmed by Davies [13]. Additionally, in his study, the trichomes were compared to the trichomes from other genera such as *Polystachya* and *Maxillaria* and significant differences were noted—“in that the pseudopollen-forming trichomes consist of a stalk cell and a ‘head’ of component cells that separate at maturity, in contrast to *Maxillaria* and some *Polystachya* spp. (…) *Maxillaria* and *Polystachya* largely contains protein, whereas and *D. unicum* the main food substances is starch” [13]. This work is of great value due to the fact that, until now, it is probably the only work analyzing the micromorphology of *Dendrobium*, and further demonstrates the morphological differences of pseudopollen compared to other orchid species. In this paper, there are the results for 21 species of *Dendrobium* presented, in particular from the nominal section.

Our studies embraced the analyses of micromorphology structures present in the labellum. Similar research was also performed for other groups of Orchidaceae, e.g., *Polystachya*, *Maxillaria*, and *Bulbophyllum*. Obviously, due to the large number of species in these genera, the topic is still unexplored. However, thanks to the results obtained by other authors, we can make a partial comparison. The analyses carried out by Teixeira et al. revealed that the most studied species of *Bulbophyllum* possess a callus, and the surface of their labellum has a papillary structure and unicellular trichomes [16]. The presence of secretions was also noted. It is worth mentioning that there are some differences in the structure of the labellum as far as the presence of osmophores and nectaries is concerned. This is related to pollination mechanisms [16]. Species of the genus *Maxillaria* have a variety of labellar structures. The hairs present on the surface may be rounded, elliptical, narrow, elongated, and fusiform, among others, depending on the species [17]. The papillae are most often cone-shaped with pointed and rounded tips, many of which are strongly pigmented [18,19]. As in *Bulbophyllum*, this is related to the diversity of visiting pollinators. Bees appear to be the main pollinators in this case, and pollination by hummingbirds has also been observed, but there is still no concrete evidence to support this [17,18]. Papillae and trichomes are also present on the surface of the labellum of *Polystachya*. The most common type of trichomes found in the analyzed species are uniseriate, two- to four-celled, with a clavate or subclavate terminal cell [8].

A recent paper examined the distribution and density of micromorphology structures on the surface of the labellum in representatives of *Polystachya* [10]. In our study, we also performed a similar analysis. In the future, based on their distribution, we can probably determine their function. Already at this stage, some fairly unambiguous conclusions can be made that the structure of the labellum and the presence of trichomes and papillae are related to pollinators. Of course, we are aware that in order to unambiguously confirm this fact and to concretely relate the distribution of structures to their role, field studies are necessary, which so far have been scarce both in *Dendrobium* and the other genera mentioned here.

Unfortunately, among *Dendrobium* species, still not much is known about pollination. Although there are a few studies on this topic, this thread remains unexplained for now. Of course, the huge number of species does not make it easier to deal with this issue. In 1988, Slater and Calder conducted a study on pollination in *Dendrobium speciosum* Sm. [20]. Unfortunately, there is a lack of micromorphological studies for this species and we do not know exactly whether, apart from typical pollinator-attracting features such as olfactory and visual exposure of flowers, whether the presence or absence of hairs and papillae on the labellum would also potentially be relevant. Based on available information, the species is known to be visited and pollinated by a variety of insects from the genera *Trigona*, *Homalictus*, *Lassioglossum*, and *Hylaeus* [20]. Studies during which species of the genus *Trigona* were determined to be pollinators were also conducted for the species *Dendrobium setifolium* Ridl. and *Dendrobium monophyllum* F.Muell. [21,22]. However, as in other cases previously, micromorphology was not analyzed. In the case of *D. monophyllum*, it is likely that bees are attracted by aromatic floral compounds. We also know that this species does not produce nectar [22]. We noted, based on studies from other groups, that often, nectar-secreting species were devoid of various ornaments, whereas, in the absence of nectar, structures on the epidermis appeared, thus suggesting the presence of a putative reward [23]. Our analyses showed that *D. monophyllum* has conical papillae. It is likely that in addition to floral compounds, these structures also contribute to attracting pollinators. Studies in the context of the pollination mechanism were also conducted for *Dendrobium infundibulum* Lindl. According to the analyses, it is mainly pollinated by *Bombus eximus*. *D. infundibulum* does not attract by scent, has no nectar, or offers any other reward to the bumblebee. Its success is based on precise pollen placement on insects as a result of a high degree of adaptation of flower morphology to the pollinator [24,25]. As in other cases, little is known about the micromorphology of this species. *Dendrobium finisterrae* Schltr. is an interesting case because certain features, such as zygomorphic flower structure, creamy green color with nectar conductors, and pleasant odor, suggest that it may be pollinated by bees. However, it also has other features, such as massive nectary spur and collenchymatous secretory tissue, indicating pollination by birds. To date, there is no clarification on this issue [26]. Another known species associated with bird pollination is *Dendrobium secundum* (Blume) Lindl. ex Wall. [27]. A very interesting phenomenon, rarely occurring among *Dendrobium*, is the self-pollination observed in *Dendrobium biflorum* (G.Forst.) Sw. on the Society Islands. This may be due to a lack of suitable pollinators or stressful growing conditions in that area. However, it is not determined whether autogamy would also occur in these taxa outside the mentioned study area [28].

The fact that structures on the labellum can be attractive to pollinators was proved by Davies by analyzing the micromorphology of *D. unicum*, which has multicellular trichomes on its labellum, as mentioned earlier [13]. This species belongs to the nominal section. In our work, we analyzed the labella of as many as seven species from this group, and every sample had micromorphology structures on the labellum, papillae, or trichomes. This may indicate two important facts: first, species in this section can also be pollinated by bees, such as *D. unicum*; second, this is an important taxonomic issue. It seems to be no coincidence that all the species of the nominal section we studied have the analyzed structures, and additionally, another taxon of this group analyzed in earlier studies has such features. This may indicate that this information has useful taxonomic value for species identification at the section level. Moreover, in 2016 a new species *Dendrobium maguanense* Q.Xu & Z.J.Liu was discovered, which is closely related to *Dendrobium crepidatum* Lindl. & Paxton. It is sometimes treated as a synonym for the latter, but the authors of the publication showed differences between the two species. Molecular analysis indicates that they belong to the section *Dendrobium* and according to the authors describing *D. maguanense*, its labellum is also covered with hairs [29]. Another species relatively recently described and included by the authors in the nominal section is *Dendrobium wenshanense* Q.Xu, Y.B.Luo & Z.J.Liu, and it also has hairs on the labellum [30].

Moreover, we were also interested in the fact that *Dendrobium alaticaulinum* P.Royen belonging similarly to *D. secundum* to the sect. *Pedilonum* (Blume) Blume has no structures on the labellum. Due to the fact that they belong to the same section we wondered if the labellum of *D. secundum*, which we mentioned earlier, is also smooth and if the lack of trichomes and papillae could be related to pollination by birds. Furthermore, as we mentioned above, there is speculation that *D. finisterrae* belonging to section *Latouria* (Blume) Miquel can be pollinated by birds as well. In our study, we analyzed the species *Dendrobium bifalce* Lindl. from this section, and interestingly, this taxon was one of the few that did not have structures on the labellum.

Of course, our assumptions should be interpreted with caution. We know that because of such a large number of species in *Dendrobium*, our sample is limited and this study is only preliminary in this broad area. Based on previous studies of other genera, it is hard to conclusively assess whether we definitely have an important taxonomic trait for *Dendrobium*. Similar studies were conducted in 2021 by Mytnik for the genus *Polystachya*, and in that case, the presence and absence of structures were important for taxonomy [10]. We are also inclined to such conclusions for *Dendrobium*, but nevertheless, take into account that this is not always the case. This is evidenced, for example, by studies conducted on the genus *Maxillaria*, in which the overall conclusions were that the papillae were of little taxonomic value in this case [31].

In our studies, we also went a step further and compared how the presence of micromorphological structures for each section is distributed on the phylogenetic trees obtained by other authors [3,5]. It is interesting to note that there is an incongruence in the topology of the tree based on the sequence of nuclear region ITS-5.8S-ITS2 and the classification proposed by Wood [4]. Numerous sections appear to be polyphyletic. We did not find correlations of the phylogenetic position of the species and the distribution of micromorphological features of the lip, either. The analyzed features’ distribution seems to respond to pollinator pressure. Based on the resulting tree (Figure 26 and Figure 27), we can confirm the above statement that the analyzed traits arose convergently. Here again, however, we must state that field studies are necessary to be certain of the above statement and also to relate our analyses of the distribution of structures on labella to their function. We realize that this is only the first step taken though, leading to very clear and interesting conclusions, which even at this stage of research on such a large genus seem highly probable.

## 4. Materials and Methods

### 4.1. Study Using Scanning Electron Microscope (SEM)

The materials used in this study came from the collections gathered at the Department of Plant Taxonomy and Nature Conservation at the University of Gdansk, Poland. All specimens were preserved in Kew Mixture consisting of 53% of ethyl alcohol, 37% of water, 5% of formaldehyde, and 5% of glycerol. They embraced 21 species of *Dendrobium*, representing 13 sections of this genus. The list of materials used is presented in Table 3. The classification is consistent with the one proposed by Wood [4].

Obtaining the appropriate photos and then drawing the correct conclusions using a scanning electron microscope required several stages of preparation of the research material. In the first step, dehydration took place, which means treating the fixed material with aqueous solutions of ethyl alcohol of increasing concentration from 25% to 100%. The dehydration procedure was carried out at room temperature, and the treatment time of the material in each of the aqueous ethyl alcohol solutions ranged from 10 to 15 min [32]. The next stage of preparations was drying the samples with the use of liquid carbon dioxide, using the occurrence of the so-called critical point using critical point dryer mark Emitech-model K850. As a result of this method, there are fewer unnecessary artifacts on the surface of the analyzed samples [33].

After that, the dried material was glued to microscope stubs, then coated with gold using Spi Module Sputter Coater. The material prepared in this way was analyzed using a Philips XL-30 (FEI) scanning electron microscope (Laboratory of Electron Microscopy, University of Gdańsk, Gdańsk, Poland) operating at an accelerating voltage of 5 or 15 kV [10].

### 4.2. Molecular Analyses

For phylogenetic reconstruction, we applied 44 sequences of the ITS region representing species of *Dendrobium* Sw. and *Cadetia* Gaudich. as outgroup taxa. Most of the sequences used in this article were downloaded from GenBank (http://www.ncbi.nlm.nih.gov). A list of the taxa with their accession numbers is included in Supplementary Materials Table S1. However, samples (leaf fragments) for 13 species of *Dendrobium* used for molecular analyses were collected in the greenhouse of the Faculty of Biology at the University of Gdansk. All sequences obtained from them were deposited in GenBank. The exact ID numbers for these samples and GenBank accession numbers are presented in Table 4.

The DNA was extracted using the DNA Sherlock AX Kit (A&A Biotechnology, Gdańsk, Poland), following the manufacturer’s protocol. Amplification and sequencing reactions were performed for nuclear region ITS1+5.8S+ITS2 using the same pair of primers, 101F and 102R [34]. The total volume of sample for PCRs was 25 µL containing 1 µL template DNA (∼10–100 ng), 0.5 µL of 10 µM of each primer, 12.5 µL StartWarm HS-PCR Mix (A&A Biotechnology, Gdańsk, Poland), and water. At the same time, the reaction parameters were taken, as Baranow [35]. The Clean-Up Concentrator Kit (A&A Biotechnology) was used to clean the PCR products following the manufacturer’s protocol. Then, the sequencing reaction was prepared and performed by Macrogen (Seoul, South Korea; http://dna.macrogen.com/eng/), using the same primers as mentioned above. All DNA sequence chromatograms were examined/edited in FinchTV (https://finchtv.software.informer.com/1.4/).

The multiple sequence files were aligned with SeaView [36] using the ‘‘align’’ option according to the MUSCLE algorithm [37]. The best fit substitution model was calculated with MrModeltest 2.2 and by both criteria [38], hLRTs (hierarchical likelihood ratio test) and AIC (Akaike information criterion) were selected GTR+G+I. For phylogenetic reconstruction, we used Bayesian inference (BI) with MrBayes v. 3.2.7a [39] and Maximum parsimony (MP) using PAUP* [40]. We used two different methods to test for possible topological incongruence.

In BI, we indicated two independent runs of four Markov-chain Monte Carlo (MCMC) chains were started from different random trees to ensure that individual runs had converged to the same result. We used 1 million generations per run with sampling every 100 generations. Convergence was assessed using the average standard deviation of split frequencies below 0.01. Thereafter, we discarded the initial 25% of the sampled generations of each chain as burn-in. Saved trees were summarized in a majority rule consensus tree. The nodal confidence was assessed by posterior probabilities (PP), which were considered strongly supported when equal to or higher than 0.95 [41].

MP analysis was undertaken with tree-bisection-reconnection (TBR) branch swapping and the MULTREES option in effect, simple addition, and ACCTRAN optimization. All characters were equally weighted [42], and missing data were coded as ‘‘?’’, and gaps as ‘‘-’’. All parsimonious trees (10,000) were used to obtain a strict consensus tree. We also performed bootstrap analysis using 1000 replicates in order to determine the internal support of clades (BS).

The BI tree was edited with FigTree v.1.4.4 (http://tree.bio.ed.ac.uk/software/figtree/) and Inkscape (https://inkscape.org/release/inkscape-1.0.2/).

## 5. Conclusions

In this study, 21 species of *Dendrobium* representing 13 sections were analyzed, including 7 species of the nominal section. The labella of all taxa in this group were covered with two different micromorphology structures. We observed cylindrical and conical trichomes, and small conical and semicircular papillae. Most species from the other sections also had conical and cylindrical trichomes and papillae, but ellipsoidal hairs and semicircular papillae were also present. In five species, the presence of the analyzed structures was not observed. This study is the first analysis in the genus *Dendrobium* focusing typically on micromorphology. Based on the micromorphological results obtained and the phylogenetic analyses performed, we suggest that the presence and absence of structures on the lips is due to convergence, and this is closely related to pollinator pressure. Of course, we recognize that confirming this thesis and accurately linking the function to the presence and distribution of structures requires an expanded study group, additional field studies, and pollinator observations. Nevertheless, the paper provides a wealth of data and a decent basis for expanding the study.

## Figures and Tables

**Table 1:** Types of labellar structures found in all *Dendrobium* species researched in this paper, which have trichomes or papillae.

Section	Species	Trichomes	Papillae
*Aporum* Blume	*Dendrobium hainanense* Rolfe	conical, cylindrical (sometimes branched)	
*Dendrobium* Sw.	*Dendrobium anosmum* Lindl.	conical, cylindrical (sometimes branched)	
	*Dendrobium brymerianum* Rchb.f.		conical, semicircular
	*Dendrobium chrysocrepis* C.S.P.Parish & Rchb.f. ex Hook.f.	conical	
	*Dendrobium nobile* Lindl.	conical, cylindrical	
	*Dendrobium parishii* H.Low	cylindrical	
	*Dendrobium regium* Prain	cylindrical, cylindrical with a bulbous cell at the apex	
	*Dendrobium scoriarum* W.W.Sm.	conical, cylindrical	
*Dendrocoryne* Lindl. & Paxton	*Dendrobium* × *delicatum* (F.M.Bailey) F.M.Bailey		conical, cylindrical, semicircular
*Formosae* (Benth. And Hk.f.) Hk.f.	*Dendrobium christyanum* Rchb.f.	ellipsoidal	
*Grastidium* (Blume) Blume	*Dendrobium katherinae* A.D.Hawkes		conical with rounded or pointed apices
*Monophyllaea* Benth.	*Dendrobium monophylum* F.Muell.		conical
*Oxyglossum* Schltr.	*Dendrobium cuthbertsonii* F.Muell.		conical, semicircular
*Rhizobium* Lindl. & Paxton	*Dendrobium mortii* F.Muell.	conical, cylindrical	
*Spatulata* Lindl.	*Dendrobium schoeninum* Lindl.	conical	conical
	*Dendrobium discolor* Lindl.		conical

**Table 2:** Relative density of trichomes and papillae on labella of *Dendrobium*.

Degree	Trichomes	Papillae	Characterization	Description as Used
4	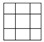	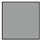	The distal parts are not visible due to the density of trichomes/papillae	Very dense
3	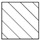	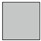	5–15 trichomes/papillae, up to 3 pieces/100 μm^2^	Dense
2	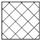	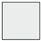	3–4 trichomes/papillae, up to 3 pieces/100 μm^2^	Rare
1	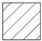	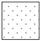	Individual trichomes/papillae, up to 3 pieces/100 μm^2^	Single/sparse
0	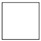	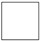	No trichomes/papillae	No trichomes/papillae

**Table 3:** List of species of *Dendrobium* used in this study (classification of the taxa follows the Wood systematic [4].

Section	Species	Accession Number
*Aporum* Blume	*Dendrobium hainanense* Rolfe	UGDA.0051616
*Conostalix* Kraenzl.	*Dendrobium attenuatum* Lindl.	UGDA.0052546
*Dendrobium* Sw.	*Dendrobium anosmum* Lindl.	UGDA.0035621
	*Dendrobium brymerianum* Rchb.f.	UGDA.0035412
	*Dendrobium chrysocrepis* C.S.P.Parish & Rchb.f. ex Hook.f.	UGDA.0116182
	*Dendrobium nobile* Lindl.	UGDA.0116184
	*Dendrobium parishii* H.Low	UGDA.0034923
	*Dendrobium regium* Prain	UGDA.0116181
	*Dendrobium scoriarum* W.W.Sm.	UGDA.0116183
*Dendrocoryne* Lindl. & Paxton	*Dendrobium* × *delicatum* (F.M.Bailey) F.M.Bailey	UGDA.0051595
*Disttichophyllae* Hk.f.	*Dendrobium uniflorum* Griff.	UGDA.0051736
*Formosae* (Benth. And Hk.f.) Hk.f.	*Dendrobium christyanum* Rchb.f.	UGDA.0052513
*Grastidium* (Blume) Blume	*Dendrobium katherinae* A.D.Hawkes	UGDA.0045354
*Latouria* (Blume) Miquel	*Dendrobium bifalce* Lindl.	UGDA.0034715
*Monophyllaea* Benth.	*Dendrobium monophyllum* F.Muell.	UGDA.0034728
*Oxyglossum* Schltr.	*Dendrobium cuthbertsonii* F.Muell.	UGDA.0034867
*Pedilonum* (Blume) Blume	*Dendrobium alaticaulinum* P.Royen	UGDA.0045288
*Rhizobium* Lindl. & Paxton	*Dendrobium mortii* F.Muell.	UGDA.0034479
	*Dendrobium schoeninum* Lindl.	UGDA.0034714
*Spatulata* Lindl.	*Dendrobium canaliculatum* R.Br.	UGDA.0035422
	*Dendrobium discolor* Lindl.	UGDA.0034720

**Table 4:** List of species of *Dendrobium* used in molecular study.

Section	Species	Accession Number	GenBank Accession Number
*Aporum* Blume	*Dendrobium bilobulatum* Seidenf.	UGDA.0076220	ON694111
*Cadetia* Gaudich.	*Cadetia quinqueloba* Schltr.	UGDA.0076230	ON694123
*Dendrobium* Sw.	*Dendrobium hekouense* Z.J.Liu & L.J.Chen	UGDA.0076247	ON694112
*Formosae* (Benth. And Hk.f.) Hk.f.	*Dendrobium williamsonii* Day & Rchb.f.	UGDA.0076204	ON694113
*Grastidium* (Blume) Blume	*Dendrobium crassifolium* Schltr.	UGDA.0076236	ON694114
*Latouria* (Blume) Miquel	*Dendrobium macrophyllum* A.Rich.	UGDA.0076205	ON694116
	*Dendrobium rigidifolium* Rolfe	UGDA.0076226	ON694115
*Oxyglossum* Schltr.	*Dendrobium brevicaule* Rolfe	UGDA.0076232	ON694117
*Pedilonum* (Blume) Blume	*Dendrobium dichaeoides* Schltr.	UGDA.0076215	ON694118
*Rhizobium* Lindl. & Paxton	*Dendrobium pugioniforme* A.Cunn. ex Lindl.	UGDA.0076149	ON694120
	*Dendrobium striolatum* Rchb.f.	UGDA.0076231	ON694119
*Spatulata* Lindl.	*Dendrobium antennatum* Lindl.	UGDA.0076155	ON694122
	*Dendrobium stratiotes* Rchb.f.	UGDA.0076213	ON694121

## Data Availability

The data presented in this study are available in Micromorphology of Labellum in Selected *Dendrobium* Sw. (Orchidaceae, Dendrobieae). However, the DNA sequences were deposited in GenBank database.

## Supplementary Materials

The supporting information can be downloaded at: https://www.mdpi.com/article/10.3390/ijms23179578/s1.
